# Epitranscriptomic signatures of m5C, m6A, and pseudouridine in COVID-19 reveal host RNA modifications involved in viral pathogenesis

**DOI:** 10.1128/spectrum.02561-25

**Published:** 2026-01-26

**Authors:** Mateusz A. Maździarz, Katarzyna Krawczyk, Ewa Lepiarczyk, Łukasz Paukszto, Jakub Sawicki, Marta Majewska

**Affiliations:** 1 Department of Botany and Evolutionary Ecology, Faculty of Biology and Biotechnology, University of Warmia and Mazury in Olsztyn 540376 , Olsztyn, Poland; 2 Department of Human Physiology and Pathophysiology, School of Medicine, Collegium Medicum, University of Warmia and Mazury in Olsztyn 175590, Olsztyn, Poland; Indian Institute of Science, Bangalore, Karnataka, India

**Keywords:** SARS-CoV-2, COVID-19, m5C, m6A, psU

## Abstract

RNA modifications represent a critical layer of post-transcriptional gene regulation, shaping RNA fate and function through modulation of splicing, stability, translation, and immune signaling. Their role in viral infections remains insufficiently explored. We applied direct RNA sequencing to profile three key RNA modifications, 5-methylcytosine (m5C), N6-methyladenosine (m6A), and pseudouridine (psU), in whole blood samples from COVID-19 patients and healthy controls. Using CHEUI and NanoSPA, we identified condition-specific modification sites, their positional distribution, and associated functions. We detected 689 m5C, 738 m6A, and 1,201 psU sites, many uniquely enriched in infected individuals. These modifications localized predominantly to 3′ UTRs and final exons. Codon-level and motif analysis revealed distinct enrichment patterns. Functional annotation indicated involvement in immune response, viral defense, inflammation, and stress. Several immune-regulatory genes, including *CSF3R*, *PIK3CD*, and *CAPZB*, exhibited co-occurring modifications. Pathway analysis revealed convergence on processes such as endocytosis, Fc receptor-mediated phagocytosis, and phagosome maturation, mechanisms central to viral entry and immune activation. Our findings show that RNA modifications are dynamically regulated during infection and may serve as biomarkers and therapeutic targets in COVID-19.

**IMPORTANCE:**

RNA modifications are increasingly recognized as critical regulators of host-virus interactions, yet their specific roles in human viral infections remain largely unexplored. Here, we provide the first comprehensive epitranscriptomic map of 5-methylcytosine (m5C), N6-methyladenosine (m6A), and pseudouridine (psU) in the host transcriptome during SARS-CoV-2 infection. By combining direct RNA sequencing with advanced modification-calling algorithms, we identify hundreds of condition-specific sites, reveal their positional preferences within transcripts, and uncover their functional association with immune defense and viral entry pathways. These findings demonstrate that host RNA modifications are dynamically remodeled during infection and define molecular signatures with potential diagnostic and therapeutic value. Our work establishes RNA modification profiling as a powerful tool for dissecting viral pathogenesis and opens new avenues for targeted antiviral strategies.

## INTRODUCTION

Epigenetic modifications, defined as heritable changes in gene expression that do not involve alterations to the underlying DNA sequence, constitute a fundamental layer of gene regulation in both physiological and pathological states. Among these alterations, RNA modifications have garnered significant attention due to their regulatory potential across diverse biological processes, as they have been identified as crucial post-transcriptional regulators of gene expression (1). Substantial progress has been made in elucidating their functional roles in modulating the processing and function of both coding and non-coding RNAs, thereby profoundly influencing diverse gene expression programs. For instance, specific RNA modifications have been observed to enhance mRNA stability, thereby augmenting its translational efficiency and leading to increased protein synthesis. Conversely, other modifications can label mRNA for degradation, thereby inhibiting its translation. Furthermore, RNA modifications play crucial roles in regulating mRNA localization within the cell and influencing alternative splicing pathways (2, 3). These modifications are integral to various biological processes, with the accurate deposition of many being essential for normal development. Conversely, dysregulation in their deposition has been implicated in numerous pathological condition (4–6).

The presence of 5-methylcytosine (m5C) in various classes of RNA, including messenger RNAs (mRNAs), transfer RNAs (tRNAs), and ribosomal RNAs (rRNAs), has been documented though its specific biogenesis, localization, and functional implications remain only partially understood (7). Equally, compelling is N6-methyladenosine (m6A), the most abundant internal modification in eukaryotic mRNA. The physiological relevance of m6A is well established, and recent studies highlight its involvement in numerous pathological processes, notably in cancer development and progression within hematopoietic, neural, and reproductive systems (8). These findings position m6A modification as a potential molecular target for therapeutic intervention. In parallel, modifications of uridine residues in RNA, such as psU, dihydrouridine, and 5-methyluridine, have emerged as additional layers of post-transcriptional control. These modifications influence RNA secondary structure and RNA–protein interactions, and several uridine-modifying enzymes have been implicated in human diseases (9).

The COVID-19 pandemic, caused by severe acute respiratory syndrome coronavirus 2 (SARS-CoV-2), has further emphasized the need for comprehensive molecular profiling of host responses to viral infection (10). Despite extensive efforts to catalog the clinical manifestations of COVID-19, a detailed understanding of the molecular determinants driving differential outcomes remains limited. The present study aims to provide an expanded view of host responses by integrating transcriptomic profiling with the investigation of key RNA modifications, namely, m5C, m6A, and psU, in whole blood samples from infected individuals. This integrative approach seeks to elucidate the epigenetic and post-transcriptional landscapes altered by SARS-CoV-2, thereby shedding light on the molecular crosstalk between viral infection, immune dysregulation, and endothelial dysfunction. Insights derived from this study hold promise for informing targeted therapeutic strategies against COVID-19 and related viral pathologies.

High-throughput complementary DNA (cDNA) sequencing technologies have significantly enhanced our understanding of transcriptome complexity and regulatory mechanisms. Nonetheless, these approaches are inherently limited by their reliance on reverse transcription and amplification, which result in short read lengths and the loss of native RNA modifications. To overcome these limitations, we employed a native direct RNA sequencing approach utilizing the platform developed by Oxford Nanopore Technologies (ONT).

## MATERIALS AND METHODS

### Sample collection and subject recruitment

Peripheral blood samples were obtained from fifteen human subjects, comprising nine healthy donors (designated 1–9) and six patients with confirmed COVID-19 (designated 10–15). The collection of blood samples from patients and controls was executed between 25 January 2021 and 15 May 2021, in alignment with institutional approval (Resolution No. 3/2021). Notably, this timeframe represented a high-transmission phase of SARS-CoV-2 in Poland, with the B.1.1.7 (Alpha) documented as the prevailing circulating variant. The COVID-19 patients were recruited from the Clinical Department of Communicable Diseases in Ostróda, Poland. Patients met the diagnostic criteria for SARS-CoV-2 infection, with viral genes confirmed via RT-PCR analysis of nasopharyngeal swab specimens. RT-PCR assays were performed using the COVID-19 Real-Time Multiplex RT-PCR Kit (Labsystems Diagnostics OY, Vantaa, Finland). This assay facilitates the simultaneous detection of the *ORF1ab*, *N*, and *E* genes within the SARS-CoV-2 genome via a single RT-PCR. RT-PCR procedures adhered strictly to the manufacturer’s prescribed protocol. Subsequent quantitative analysis of amplification curves was performed utilizing a QuantStudio 5 Real-Time PCR System. Inclusion criteria for this study mandated a confirmed positive SARS-CoV-2 PCR test coupled with a clinical diagnosis of COVID-19 necessitating hospital admission. Patients were excluded based on the presence of neoplasia, autoimmune disorders, immunosuppression, immunodeficiency, or human immunodeficiency virus (HIV) infection. The control group consisted of healthy volunteers who tested negative for SARS-CoV-2 and exhibited no clinical signs of respiratory tract infections or pulmonary pathologies, as confirmed by a medical professional. The control group was established based on the following inclusion criteria, confirmed via a screening questionnaire: no history of travel to high-risk areas, no SARS-CoV-2 vaccination, no documented or suspected SARS-CoV-2 exposure within the preceding 14 days, absence of active upper or lower respiratory tract infection or any other acute illness at the time of blood collection, and no history of severe chronic diseases, including immune disorders.

The patients diagnosed with SARS-CoV-2 infection, comprised three females and three males, aged between 54 and 70 years. All patients underwent chest computed tomography (CT) imaging, which demonstrated various degrees of pulmonary involvement ranging from 10% to 90% of the lung parenchyma. Radiological findings included ground-glass opacities, consolidations, fibrotic strands, and in severe cases, extensive bilateral inflammatory changes typical for COVID-19 pneumonia. Mild pleural effusion was observed in some patients, and mediastinal lymphadenopathy was present in advanced cases. Biochemical analysis revealed variable abnormalities. Alanine aminotransferase (ALT) levels ranged from 4 to 147 U/L (norm 0–41), and aspartate aminotransferase (AST) from 10 to 85 U/L (norm 0–40), with four patients showing mild to moderate elevation. C-reactive protein (CRP) was increased in all cases (6.5–107 mg/L; norm 0–5), correlating with disease severity. D-dimer levels varied between 0.57 and 548.6 ng/mL, indicating differing degrees of coagulation activation. White blood cell counts ranged from 4.59 × 10³/µL to 8.9 × 10³/µL, remaining mostly within normal limits. Overall, the laboratory findings reflected systemic inflammation and coagulopathy of varying intensity, consistent with the clinical spectrum of COVID-19 (Table 1).

**TABLE 1:** Clinical characteristics of study participants^*a*^

Patient no.	Gender	Age	Lung involvement (CT)	Chest computed tomography description	ALT U/L [0–41]	AST U/L [0–40]	CRP mg/L [0–5]	D-dimers ng/mL	WBC × 10³/µL
COV-19 P1	F	64	20%–40%	Inflammatory infiltrates in the parenchyma of both lungs, probably due to the underlying disease, predominantly in the lower fields bilaterally	10	28	65.4	548.6	6.66
COV-19 P2	M	70	15%	Mild inflammatory infiltrates in the lung parenchyma bilaterally, predominantly in the right lung, probably due to the underlying disease	147	76	6.5	2.75	4.59
COV-19 P3	F	67	10%–20%	Ground-glass opacities with foci of consolidation involving about 10%–20% of the parenchyma. Single fibrotic strands. Trace amounts of pleural fluid (up to 5 mm) in both pleural cavities	38	44	33.8	1.02	7.1
COV-19 P4	M	69	10%–20%	Ground-glass opacities with foci of consolidation involving about 10%–20% of the parenchyma. Single fibrotic strands. Trace amounts of pleural fluid (up to 5 mm) in both pleural cavities. No enlargement of mediastinal or hilar lymph nodes	43	85	44.2	34.74	5.5
COV-19 P5	F	90	90%	In both lungs, areas of ground-glass opacity with interlobular septal thickening are visible—differential diagnosis includes pulmonary edema and interstitial inflammatory changes associated with COVID-19 infection (involving about 90% of lung parenchyma). Atelectasis/atelectatic-inflammatory changes in the lower lobes. Pleural fluid present: up to ~5 cm thick layer on the right and ~2 cm on the left. Enlarged mediastinal lymph nodes up to ~16 × 11 mm	33	70	28	3.18	8.9
COV-19 P6	M	64	75%–80%	Lungs with markedly reduced aeration; in all lobes, there are confluent areas of ground-glass and “crazy paving” pattern, with additional patchy parenchymal consolidations—most prominent in the upper lobe of the left lung. The image corresponds to severe inflammatory changes typical of COVID-19. About 70%–75% of the right lung and 75%–80% of the left lung parenchyma are affected. No pathological enlargement of mediastinal lymph nodes. Trachea and major bronchi with normal lumen. Pleural cavities free of fluid	4	10	107	0.57	7.6

^*a*^ The table presents demographic and clinical data for the study groups, including six COVID-19 patient cohorts (COV-19 P1–P6), categorized according to the extent of pulmonary involvement and the need for respiratory support. The percentage of pulmonary involvement was assessed by chest computed tomography. Biochemical analysis values included alanine aminotransferase (ALT), aspartate aminotransferase (AST), C-reactive protein (CRP), D-dimer levels, and white blood cell counts (WBC). For each participant, sex (F, female; M, male) and age (years) are reported.

Whole blood samples, 3 mL in volume, were acquired from each patient and immediately transferred to Tempus Blood RNA Tubes (Applied Biosystems, Waltham, Massachusetts, USA). Samples were subsequently stored at −80°C until subsequent analysis.

### Total RNA extraction from peripheral blood

Total RNA was extracted from whole blood samples obtained from both experimental and control groups utilizing the Tempus Spin RNA Isolation Kit (Applied Biosystems, Waltham, MA, USA). Total RNA was extracted from whole blood and subjected to poly(A) magnetic bead-based enrichment prior to direct RNA sequencing. This approach was selected based on the Nanopore protocol requirements, which rely on polyadenylated transcripts for adapter ligation. As a result, the sequencing predominantly captured host polyadenylated RNAs. Due to the low abundance of SARS-CoV-2 RNA in blood samples and the nature of the enrichment strategy, viral RNAs were not sufficiently detected for modification analysis. Prior to extraction, the frozen blood samples, stored in Tempus tubes, were thawed and quantitatively transferred to 50 mL conical tubes. Subsequently, 3 mL of PBS, devoid of Ca²^+^ and Mg²^+^, was added to each tube, bringing the total volume to 12 mL. Samples were then subjected to vigorous vortexing for a minimum duration of 30 s, followed by centrifugation at 3,000 × *g* for 30 min at 4°C. The supernatant was decanted, and the resulting RNA pellet was purified in accordance with the manufacturer’s protocol. The quantity and integrity of the extracted total RNA were subsequently assessed using an Agilent 2100 Bioanalyzer (Agilent Technologies, USA).

### Nanopore direct RNA sequencing

Total RNA isolates underwent mRNA enrichment via the NEBNext Poly(A) mRNA Magnetic Isolation Module (New England Biolabs) to deplete rRNA. Subsequently, long-read libraries were prepared from 50 ng of poly(A)-tailed mRNA per sample utilizing the Direct RNA Sequencing Kit SQK-RNA002 (Oxford Nanopore Technologies), adhering to the manufacturer’s guidelines. The initial step of library preparation involved the synthesis of a complementary DNA (cDNA) strand to the RNA, forming an RNA-cDNA hybrid, catalyzed by SuperScript III Reverse Transcriptase (Thermo Fisher Scientific). Subsequently, sequencing adapters were ligated using T4 DNA Ligase (2M U/mL, New England Biolabs) in conjunction with NEBNext Quick Ligation Reaction Buffer. Libraries were quantified using the Qubit dsDNA HS Assay Kit (ThermoFisher) and subjected to sequencing on a MinION MK1C sequencing device (ONT) with FLO-MIN 106 Flow Cells R.9.4.1 (ONT). Flow Cells were prepared for sequencing using the Flow Cell Priming Kit EXP-FLP002 (ONT). Raw MinION signals, initially in POD5 format, were converted to FAST5 format using the pod5-file-format program (https://github.com/nanoporetech/pod5-file-format). Finally, transcriptomic sequences were basecalled employing Guppy v.6.0.0 (https://community.nanoporetech.com/docs/prepare/library_prep_protocols/Guppy-protocol/v/gpb_2003_v1_revax_14dec2018/guppy-software-overview).

### 5-Methylcytosine (m5C) and N6-methyladenosine (m6A) sites detection

The FAST5 files were combined to enable the re-execution of basecalling for both the COVID-19 and control groups. The resulting FASTQ files were re-mapped to the transcriptome sequence (*H. sapiens* v.GRCh38) using the minimap2 v.2.26 software with the *-ax map-ont* parameter. The resulting BAM files were then sorted using samtools v.1.16.1 (11). Signal data were rescaled to the aligned sequences using nanopolish. Differential m5C and m6A modification information was extracted using the CHEUI tool (https://github.com/comprna/CHEUI). This preprocessing involved predicting stoichiometry values and modification probabilities at transcriptomic sites via two distinct models. Genes encoding hemoglobin were subsequently excluded from further analysis. Next, sites that occurred in both control and COVID patients were subjected to differential analysis, in which only sites with *P*-value < 0.05 and the absolute value of stoichiometry differentiation (stoichiometry diff) > 0.1 were deemed statistically significant. Positions were assigned to exons, codons, and transcript annotations using a GTF file and the GRCh38.110 transcriptome.

### Pseudouridine site detection

The FASTQ files, encompassing data from both COVID-19 and healthy patient cohorts, were divided into separate data sets, after which psU site identification was conducted using the NanoSPA tool (https://github.com/sihaohuanguc/NanoSPA). The reference transcriptome, derived from long-read sequencing data, was analyzed using the remove_intron, extract_features, and prediction_psU functions. All analyses were performed using default parameter settings. Pseudouridine sites exhibiting a probability exceeding 0.95 were deemed statistically significant. Positions located within hemoglobin-encoding genes were excluded from the analysis. The assignment of positions to exons, codons, and transcript annotations was conducted with the aid of a GTF file and the GRCh38.110 transcriptome.

### Functional annotations

All molecules exhibiting reliably detected modifications were subsequently analyzed for enrichment in Gene Ontology (GO) annotations (12, 13) using the g:profiler v.0.2.2 R package (14). GO processes with an adjusted *P*-value of less than 0.05 were considered statistically significant. The enrichment *z*-score was calculated using the GOplot v.1.0.2 library (15).

### Visualization

The visualizations were generated using the R environment and the following packages: ggplot2 v.3.5.1 (16), ComplexHeatmap v.2.18.0 (17), ggseqlogo v.0.2 (18), and ggvenn v.0.1.10 (https://github.com/yanlinlin82/ggvenn).

## RESULTS

### The impact of pseudouridine modifications on RNA in COVID-19

**Fig 1:**
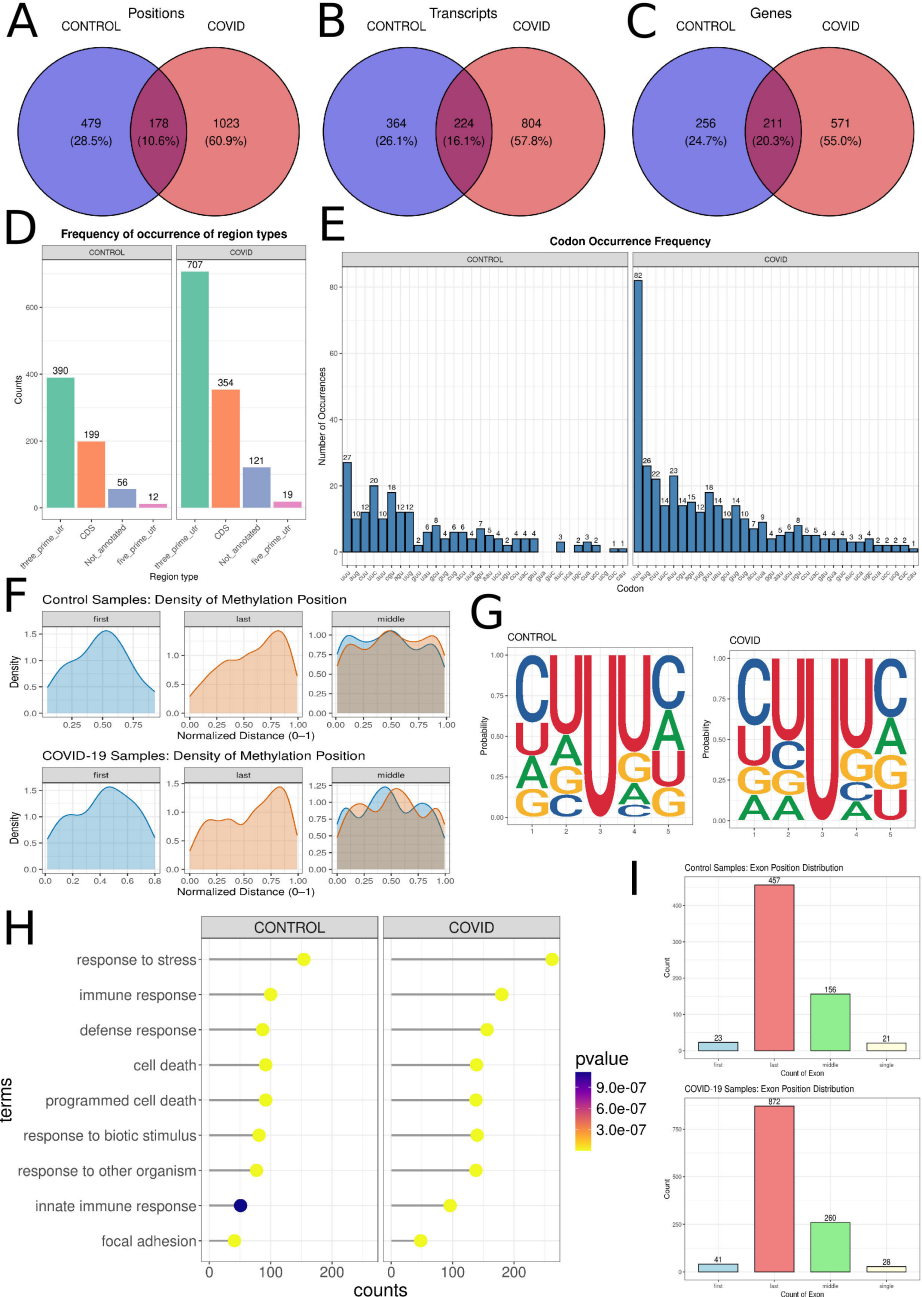
Distribution of pseudouridine (psU) in the control and COVID-19 samples. (**A–C**) Venn diagrams illustrate the occurrence of psU in the control group (blue), COVID-19 group (red), and the overlap between the two groups (dark red) for (**A**) positions, (**B**) transcripts, and (**C**) genes. (**D**) The barplots illustrate the frequency of psU within transcript fragments. The left side represents control samples, while the right side shows COVID-19 samples. Green bars indicate the 3′-UTR, orange bars represent the CDS, blue bars denote not annotated regions, and pink bars show the 5′-UTR. (**E**) The blue barplots display the assignment of psU to codons. The height of each bar indicates frequency, and the *x*-axis represents the codons. The left panel presents data from control samples, while the right panel displays data from COVID-19 samples. (**F**) The density plot illustrates the distances from the start of the exon (blue) and to the end of the exon (orange) in the first, last, and middle exons. (**G**) The figure illustrates the psU-containing motif at the third position for both control and COVID-19 samples. (**H**) The lollipop plot depicts significantly enriched GO processes. Control samples are displayed on the left panel, while COVID-19 samples are presented on the right panel. The *y*-axis enumerates the specific GO processes, and the *x*-axis quantifies the number of transcripts associated with each process. The color of each data point (dot) indicates the statistical significance (*P*-value) of the enrichment, with the corresponding scale provided on the right. (**I**) The barplots display the distribution of psU positions within exons. Blue represents the first exon, red represents the last exon, green represents middle exons, and beige represents single exons.

Pseudouridine was identified as the most prevalent post-transcriptional modification in RNA. A total of 1,201 statistically significant psU modification sites were identified in RNA samples from COVID-19 patients, compared to 657 sites in control subjects. Of these, 178 positions were common to both cohorts (Fig. 1A; Tables S1 and S2). These modifications were distributed across 1,028 distinct transcripts in COVID-19 patients and 588 transcripts in controls (Fig. 1B; Tables S1 and S2). Analysis at the gene level revealed psU modifications within 782 genes in COVID-19 patients and 467 genes in control individuals (Fig. 1C; Tables S1 and S2). Positions were assigned to transcript regions. In the control group, 390 positions were attributed to the 3′-UTR, 199 to the CDS, 12 to the 5′-UTR, and 56 to unannotated regions. Conversely, in COVID-19 patients, 707 positions were assigned to the 3′-UTR, 354 to the CDS, 121 to unannotated regions, and 19 to the 5′-UTR (Fig. 1D; Table S3). Within CDS regions, psU was most frequently detected within the UUU codon, accounting for 27 instances in the control group and 82 in the COVID-19 group (Fig. 1E; Table S4). The distribution of psU within exons also demonstrated distinct patterns: ψ sites on the first exon were predominantly localized towards the middle, while those on the last exon were situated closer to the exon start. Pseudouridine within middle exons displayed a more diffuse distribution throughout the entire exon (Fig. 1F). In the AUG codon, 10 psU were detected in control samples and 26 in COVID-19 samples. Similarly, in the CUU codon, 12 psU were identified in control samples and 22 in COVID-19 samples (Fig. 1E; Table S4). Pseudouridine modifications were identified in 21 monoexonic transcripts in control samples and in 28 in COVID-19 samples (Fig. 1I). Regarding specific exons, psU were detected in the following quantities: 23 in the first exon in control samples and 41 in COVID-19 samples, 457 in the last exon in control samples and 872 in COVID-19 samples, and 156 in middle exons in control samples and 260 in COVID-19 samples (Fig. 1I). In both control and COVID-19 samples, the consensus five-nucleotide sequence observed was CUUUC, with cytosine at the first and fifth positions, and uracil at the second and fourth positions. Pseudouridine was consistently identified at the third position (Fig. 1G). GO enrichment analysis of genes exhibiting statistically significant psU revealed distinct biological processes in the COVID-19 and control groups. In the COVID-19 cohort, prominent GO terms associated with these genes included immune response (GO:0006955), response to virus (GO:0009615), and defense response (GO:0006952), all of which were statistically significant (Fig. 1H; Table S5). Conversely, in the control group, while immune response (GO:0006955) and defense response (GO:0006952) were also identified as significant processes, the response to virus (GO:0009615) term was notably absent (Fig. 1H; Table S6).

### Comparative analysis of 5-methylcytosine (m5C) methylation between control and COVID-19 groups

**Fig 2:**
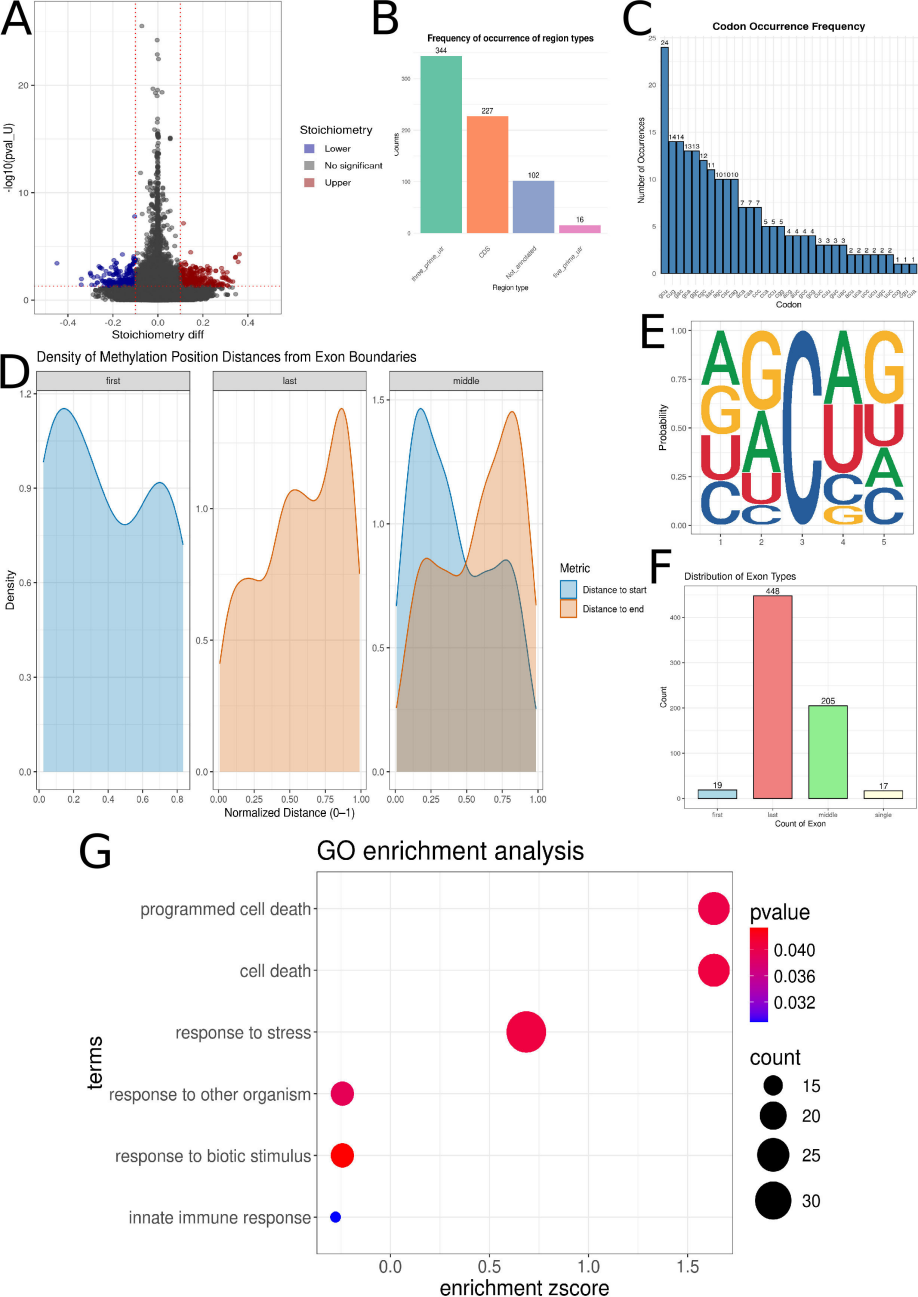
Distribution of (5-methylcytosine) m5C methylation in control and COVID-19 samples. (**A**) The Volcano plot illustrates the relationship between m5C stoichiometry and *P*-value (pval_U) at specific modification sites. The *y*-axis displays the negative base-10 logarithm of the *P*-value [−log₁₀(pval_U)], while the *x*-axis represents the difference in stoichiometry. Positions with a stoichiometry difference less than −0.1 are marked in blue, and those with a stoichiometry difference greater than 0.1 are marked in red. (**B**) The bar plots show the frequency of m5C within transcript fragments. Green bars indicate the 3′-UTR, orange bars represent the CDS, blue bars denote unannotated regions, and pink bars show the 5′-UTR. (**C**) The blue bar plots illustrate the assignment of m5C to specific codons. Each bar’s height quantifies the frequency of m5C occurrence within a given codon, with codons represented along the *x*-axis. (**D**) The density plot illustrates the distances from the start of the exon (blue) and to the end of the exon (orange) in first, last, and middle exons. (**E**) The figure depicts the m5C-containing motif at the third position. (**F**) The bar plots show the distribution of m5C positions within exons. Blue represents the first exon, red the last exon, green middle exons, and beige single exons. (**G**) The plot shows significant GO processes. The *y*-axis is designated for the specific GO processes identified. The *x*-axis quantifies the enrichment *z*-score corresponding to the stoichiometric differences observed within each process. The magnitude of the dot directly correlates with the number of transcripts associated with a given GO process. Furthermore, the color of each dot signifies the *P*-value of the enrichment, with the corresponding scale provided on the right for interpretation.

The CHEUI program enabled a comparison of m5C methylation sites between the control and COVID-19 groups. The analysis identified 689 statistically significant m5C methylation positions across 398 transcripts, corresponding to 161 unique genes. Of these, 323 positions exhibited a stoichiometry_diff < 0.1 (Lower), while 366 were found to exhibit stoichiometry_diff > 0.1 (Higher) (Fig. 2A; Table S7). Significant positions were assigned to regions within transcripts: 344 to the 3′-UTR, 227 to the CDS, 102 to Not_annotated regions, and 16 to the 5′-UTR (Fig. 2B; Table S3). The positions were most frequently found in GCU codons (19), CUG codons (14), and GAC codons (14) (Fig. 2C; Table S4). M5C methylations detected in the first exon (20) were predominantly located at the beginning of the first exon. Those detected in the last exon (448) were predominantly found at the end of the exon, while those found in middle exons (205) were also predominantly located at the beginning of the exon (Fig. 2D and F). m5C was most frequently observed to be accompanied by adenine at the first position, guanine at the second position, adenine at the fourth position, and guanine at the fifth position within a 5-nucleotide motif where the methylated cytosine was located at the third position (Fig. 2E). Significant processes such as cell death (GO:0008219), response to stress (GO:0006950), and innate immune response (GO:0045087) were revealed by GO analysis (Fig. 2G; Table S8).

### Comparative analysis of N6-methyladenosine (m6A) methylation between control and COVID-19 groups

**Fig 3:**
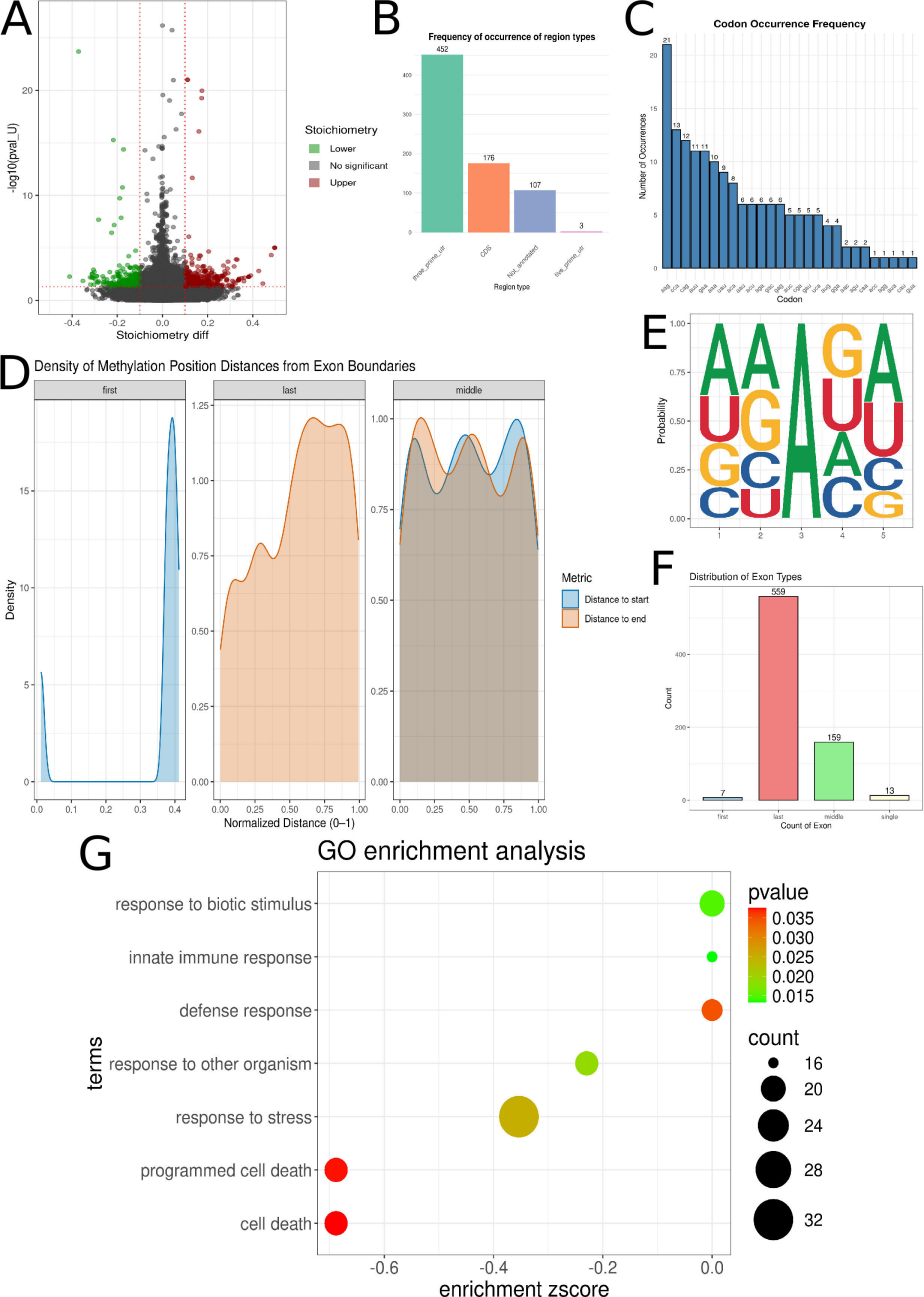
The distribution of N6-methyladenosine (m6A) methylation in control and COVID-19 samples. (**A**) The Volcano plot illustrates the relationship between m6A stoichiometry and *P*-value (pval_U) at specific modification sites. The *y*-axis displays the negative base-10 logarithm of the *P*-value [−log₁₀(pval_U)], while the *x*-axis represents the difference in stoichiometry. Sites with a stoichiometry difference below −0.1 are highlighted in green, whereas those above 0.1 are shown in red. (**B**) Bar graphs display the frequency of m6A within distinct transcript regions. The 3′-UTR is represented by green bars, the CDS by orange bars, unannotated regions by blue bars, and the 5′-UTR by pink bars. (**C**) The blue bar graphs quantitatively illustrate the codon distribution of m6A. The frequency of m6A occurrence within each specific codon is represented by the height of its corresponding bar, with all codons systematically arranged along the *x*-axis. (**D**) Exon distances, measured from both the start (blue) and end (orange) of the exon, are visualized as density distributions across first, last, and internal exons. (**E**) The figure identifies the m6A-containing motif at the third nucleotide position. (**F**) The distribution of m6A sites within exons is presented in the bar graph. Blue signifies the first exon, red indicates the last exon, green marks middle exons, and beige denotes single exons. (**G**) The plot highlights statistically significant GO processes. GO processes are listed on the *y*-axis, and the *x*-axis quantifies the enrichment *z*-score of the stoichiometry difference for each process. The size of each point reflects the total number of transcripts involved, and the point color indicates the *P*-value, referencing the scale on the right.

A total of 169,359 positions were detected across control and COVID-19 samples, of which 738 were statistically significant. The statistically significant m6A modifications were assigned to 414 transcripts within 157 genes. Of these, 287 positions were observed to have a stoichiometry less than −0.1, while 451 had a stoichiometry greater than 0.1 (Fig. 3A; Table S9). The m6A positions were classified into various regions: 452 in the 3′ UTR, 176 in the CDS, 107 as not annotated, and 3 in the 5′ UTR (Fig. 3B; Table S3). These were further assigned to codons such as AAG (21), CCA (13), and CAG (12) (Fig. 3C; Table S4). Regarding their distribution within exons, the m6A sites detected in the first exon (7) were primarily located in the middle, whereas those detected in the last exon (559) were found at the beginning. Conversely, m6A sites identified in middle exons (159) were distributed in similar proportions throughout the entire exon (Fig. 3D and F). Within the 5-nucleotide motif, the methylated adenine was most frequently observed at the third position. This motif predominantly featured adenine at the first, second, and fifth positions, with guanine at the fourth (Fig. 3E). Genes containing m6A modifications were found to be involved in processes such as defense response (GO:0006952), response to stress (GO:0006950), cell death (GO:0008219), and innate immune response (GO:0045087) (Fig. 3G; Table S10).

### The profiling of modified genes and KEGG pathway association

**Fig 4:**
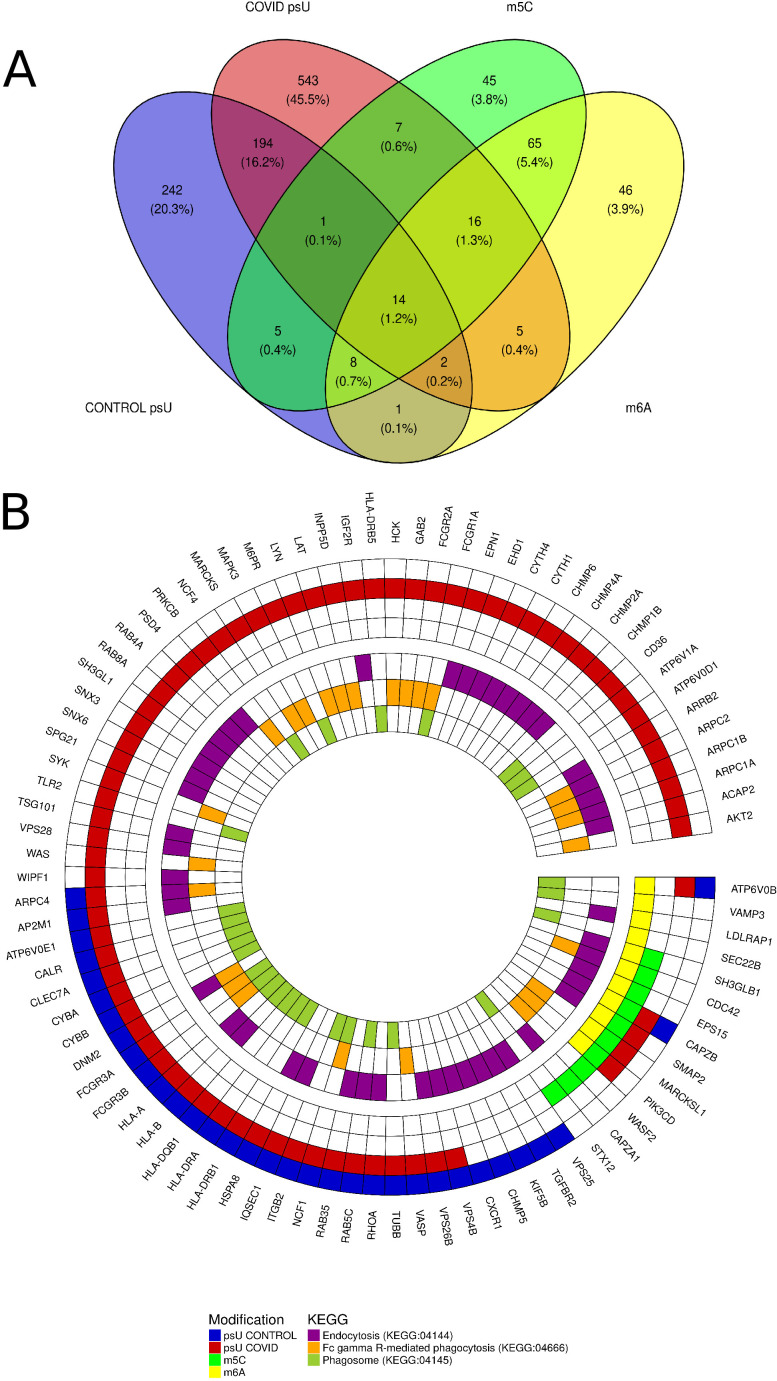
The distribution of RNA modifications and associated functional pathways. (**A**) Venn diagram illustrates the overlap in the occurrence of epitranscriptomic modifications of pseudouridine (psU) in the control group (blue) and in the COVID-19 group (red), together with 5-methylcytosine (m5C) (green), and N6-methyladenosine (m6A) (yellow) modifications. (**B**) A circular plot depicts genes participating in KEGG pathways. The outer track displays gene names. The first heatmap illustrates the presence of modifications within each gene across the control psU group (blue), COVID-19 psU group (red), m5C (green), and m6A (yellow). The inner heatmap illustrates the association of genes with the enriched KEGG pathways: “Endocytosis” (KEGG:04144) (purple), “Fc gamma R-mediated phagocytosis” (KEGG:04666) (orange), and “Phagosome” (KEGG:04145) (green).

Modifications were detected in 1,194 genes. Of these, all modifications were identified in 14 specific genes: *CAPZB*, *RHOC*, *WDTC1*, *THEMIS2*, *LCK*, *STK40*, *TXNDC12*, *CSF3R*, *PTAFR*, *ZDHHC18*, *LYPLA2*, *FAAP20*, *SMIM12*, and *S1PR1* (Fig. 4A). All genes with detected modifications were assigned to KEGG pathways. Significant pathways were found to be “Endocytosis” (KEGG:04144), “Fc gamma R-mediated phagocytosis” (KEGG:04666), and “Phagosome” (KEGG:04145) (Fig. 4B; Table S11).

## DISCUSSION

### The impact of SARS-CoV-2 infection on 5-methylcytosine (m5C) dynamics

The methylation of the cytosine residue at position 5 represents a ubiquitous RNA modification, primarily catalyzed by members of the NOL1/NOP2/SUN domain (NSUN) family, specifically NSUN1 through NSUN7. Among these enzymes, NSUN2 exhibits particularly broad substrate specificity, underscoring its significant role in the cellular landscape of m5C modification. This modification has been demonstrated to be integral to various facets of RNA metabolism, including tRNA stability, rRNA assembly, and mRNA translation (7).

Observations indicate that viral infections exert a discernible influence on m5C modification processes. For instance, bisulfite sequencing analyses have revealed that infection with simian retrovirus induces alterations at 2,475 m5C sites located within 517 genes pertinent to viral infection (20). Despite these findings, the precise functional implications of m5C modification in the context of viral biology remain largely undefined, representing a significant area for future investigation.

The genomic RNAs (gRNAs) of SARS-CoV-2 carry abundant m5C modifications that affect the viral life cycle (21, 22). Notably, a reduction in m5C levels in viral RNAs, resulting from the depletion of *NSUN2*, appears to enhance viral replication and infectivity. Furthermore, NSUN2-mediated m5C modifications across different regions of viral transcripts lead to decreased stability and reduced levels of the corresponding mRNAs. This suggests that *NSUN2* acts as a crucial host restriction factor against SARS-CoV-2 infection, as evidenced by the more severe infection and lung damage observed in NSUN2-deficient mice compared to control mice (23). Collectively, these findings underscore the critical role of m5C modifications in SARS-CoV-2 replication and pathogenicity. The present investigation identified 689 m5C methylation sites in SARS-CoV-2-infected cells, assigned to the T cell receptor signaling pathway and alpha-beta T cell activation. Similarly, Jiang et al. (24) utilized methylated RNA immunoprecipitation sequencing to profile m5C modifications in lncRNAs in H1N1 influenza A virus-infected and uninfected A549 cells. Their study identified 2,984 differentially modified m5C sites on lncRNAs in influenza A virus-infected cells, with 1,317 sites upregulated and 1,667 downregulated compared to the uninfected control. Moreover, the target genes of these methylated lncRNAs were significantly enriched in pathways relevant to various viral infections, including Epstein-Barr virus, measles, and herpes simplex virus 1.

In the present study, among the analyzed genes, *CSF3R*, *CSDE1*, and *KCNAB2* were found to be most enriched with m5C modifications. *CSF3R*, an inflammation-related gene, plays a role in the immune response to SARS-CoV-2 infection and has been found to be significantly upregulated in the blood immune cells of COVID-19 patients (19). Moreover, it acts as a receptor that promotes the activation of granulocytes and macrophages, subsequently leading to an increase in inflammatory cytokine production (25). *CSDE1* has been demonstrated to be essential for translation in human rhinovirus and poliovirus (26). SARS-CoV-2, in its strategy to succeed, relies on its capacity to co-opt host RNA-binding proteins (RBPs) while evading antiviral RBPs. Transcriptomic analyses have identified *CSDE1* as a proviral RBP that influences multiple stages of the mRNA lifecycle. Suppression of *CSDE1* expression has been shown to inhibit SARS-CoV-2 replication by reducing viral RNA levels (27). Furthermore, SARS-CoV-2 may recruit *CSDE1* to regulate internal ribosomal entry site (IRES)-dependent translation initiation of both SARS-CoV-2 gRNA and subgenomic RNAs (sgRNAs) (28). Finally, *KCNAB2*, a regulatory beta subunit of potassium voltage-gated channels, was identified as an interferon-stimulated gene specifically upregulated in response to type I interferon-mediated antiviral signaling upon SARS-CoV-2 infection (29).

### Altered N6-methyladenosine (m6A) RNA modification landscape in response to SARS-CoV-2 infection

N6-methyladenosine modification has been recognized as a key post-transcriptional regulatory mark across various classes of RNA, including mRNAs, tRNAs, rRNAs, circular RNAs (circRNAs), microRNAs (miRNAs), and long non-coding RNAs (lncRNAs) (30). Moreover, m6A modification has been demonstrated to play critical roles in the regulation of RNA splicing, translation, stability, subcellular localization, and secondary structure (31).

The present study detected 169,359 m6A modification sites in control and COVID-19 samples, out of which 738 were statistically significant, corresponding to 414 transcripts across 157 genes. These modifications exhibited varying stoichiometry, with the majority located in the 3′ UTR and fewer in coding regions, 5′ UTR, or unannotated regions. Functionally, genes with significant m6A changes were enriched in pathways related to immune response, stress response, and cell death.

Among the genes identified as bearing m6A modifications in the present study, several—including *CDC42, PIK3CD, ATP6V0B*, *SEC22B*, and *CAPZB*—appear to play particularly critical biological roles. Notably, we found that *PIK3CD* and *CAPZB* exhibited not only m6A modifications but also m5C and psU modifications, suggesting a complex layer of epitranscriptomic regulation. *CDC42* has been implicated in SARS-CoV-2 spike protein-induced cellular senescence. Nong et al. (32) demonstrated that the SARS-CoV-2 spike protein accelerates cellular aging by upregulating CDC42 expression, which subsequently activates the Wnt/β-catenin signaling pathway. Conversely, pharmacological inhibition of CDC42 with ML141 in animal models mitigated cellular senescence, lung injury, and inflammation, suggesting that spike protein-mediated CDC42 upregulation drives cellular senescence and enhances β-catenin nuclear translocation. *PIK3CD* is critical for proper immune function, with defects leading to immunodeficiency and increased susceptibility to severe infections. Potts et al. (33) described a rare case of COVID-19-related encephalitis in a patient with an inborn error of immunity resulting from a *PIK3CD* defect. This correlation between *PIK3CD* alterations and immunodeficiency has been independently corroborated by other studies (34, 35). Furthermore, *ATP6V0B* plays a role in regulating the immune response by influencing the acidification of intracellular compartments and vesicular trafficking, processes essential for viral entry and replication. Inhibition of V-ATPase function, to which *ATP6V0B* contributes, has been shown to restrict viral replication, highlighting its importance in the viral lifecycle (36). The *SEC22B* gene also encodes a protein strongly engaged in the immune response. SEC22B is found at phagosomes (37) which are crucial for host defense and tissue homeostasis, indicating its involvement in phagocytic processes. Finally, *CAPZB* has been previously identified as a potential therapeutic target during COVID-19 pathogenesis (38).

### Pseudouridylation dynamics in SARS-CoV-2 infection: implications for immune modulation and RNA vaccine efficacy

The present study also investigated psU, which is one of the most abundant post-transcriptional modifications of RNA (39). The analysis identified a total of 1,201 statistically significant psU modification sites in patients infected with SARS-CoV-2, compared to 657 such sites in control individuals, with 178 sites common to both cohorts. The GO analysis of genes with statistically significant *P*-values revealed that the COVID-19 group was significantly enriched for biological processes related to immune response, response to virus and defense response once again.

Pseudouridylation has been implicated in modulating translation efficiency and cellular stress responses. Previous studies have linked dysregulation of pseudouridylation with the pathogenesis of various cancers and genetic disorders (40). The enhanced translational rates observed for pseudouridylated mRNAs are thought to result from the reduced activation of RNA-dependent protein kinase, a phenomenon more frequently triggered by unmodified mRNA (40, 41).

Notably, the pseudouridylation profile observed in SARS-CoV-2-infected patients aligns closely with that reported in HeLa cells under similar conditions. Furthermore, the deliberate incorporation of N1-methyl-pseudouridine (m1psU) modifications into mRNA vaccines has proven pivotal in reducing innate immunogenicity, thereby enhancing the stability and translational capacity of mRNA vaccines developed against COVID-19 (40, 41). Consistent with this, Izadpanah et al. (42) proposed that SARS-CoV-2 may exploit host RNA pseudouridylation mechanisms as a strategy to evade immune detection.

### Comparative analysis of epitranscriptomic signatures across RNA viruses

To situate our results within the broader viral-epitranscriptome literature, we compared the present m5C, m6A, and psU signatures with published data sets from other RNA viruses. Alterations in m6A have been repeatedly observed across diverse viral infections, for example, HIV-1, showing virus-induced remodeling of host and viral m6A landscapes that influence viral replication and host immune signaling (43). Similarly, transcriptome-wide m5C rearrangements were reported in A549 cells infected with H1N1 influenza A virus, with differentially methylated sites enriched in pathways relevant to antiviral defense, which parallels our observation of m5C enrichment among immune-related genes (24). Pseudouridylation changes have also been mapped in both host and viral RNAs and implicated in modulating translation and innate immune recognition; antibody- and crosslinking-based psU mapping methods demonstrate that psU dynamics accompany viral infection and can affect host-pathogen interactions (44). Finally, recent functional work implicates NSUN2-mediated m5C as a host factor that restricts SARS-CoV-2 replication, consistent with our identification of m5C-enriched immune genes (22). Together, these comparative data indicate that the epitranscriptomic shifts we report are not unique to SARS-CoV-2 but instead reflect conserved host responses and viral strategies across distinct RNA viruses; this strengthens the generalizability of our pathway-level conclusions while highlighting candidate modification sites for future mechanistic study.

### Mapping RNA 5-methylcytosine (m5C), N6-methyladenosine (m6A), and pseudouridine (psU) modifications and their KEGG pathway associations in SARS-CoV-2

The current investigation has unveiled a critical association between modified genes and fundamental cellular processes. Our analysis, mapping these genes to the KEGG pathways, identified several highly significant enrichments. Among these, the most prominent were “Endocytosis (KEGG:04144),” “Fc gamma R-mediated phagocytosis (KEGG:04666),” and “Phagosome (KEGG:04145).

SARS-CoV-2 utilizes host endocytic pathways for cellular entry, exploiting clathrin-mediated endocytosis following binding of its spike (S) glycoprotein to the angiotensin-converting enzyme 2 (ACE2) receptor (45). This internalization process is dependent on several key endosomal regulators, such as PIKFYVE, which modulates endosomal maturation and trafficking, thereby facilitating the transport of the virus into acidic compartments necessary for viral uncoating and fusion (46)

Parallel to this entry mechanism, the host immune system engages via Fc gamma receptor (FcγR)-mediated phagocytosis. Specifically, the opsonization of viral particles by immunoglobulin G (IgG) promotes their recognition by FcγR-expressing phagocytes, including monocytes and macrophages. Binding of immune complexes to FcγRIIA (CD32A) initiates a signaling cascade involving Src family kinases, Syk, and downstream actin cytoskeleton rearrangement, culminating in phagocytic uptake (47). This process, while contributing to viral clearance, has been implicated in antibody-dependent enhancement (ADE), a phenomenon whereby FcγR-mediated uptake of opsonized virus leads to increased infection or immune activation in certain immune cells (48). Following internalization, the formation and maturation of phagosomes are integral to antigen degradation. Phagosomes sequentially fuse with lysosomes to form phagolysosomes, where the acidic environment and hydrolases facilitate the degradation of viral components. This process also promotes the generation of reactive oxygen species and presentation of antigens via major histocompatibility complex (MHC) molecules (49). Importantly, SARS-CoV-2 can infect mononuclear phagocytes, either through ACE2-dependent pathways or via FcγR-mediated uptake, often resulting in abortive infection characterized by inflammasome activation and pyroptotic cell death. This contributes to the release of pro-inflammatory cytokines such as interleukin-6 (IL-6), interleukin-1β (IL-1β), and tumor necrosis factor-alpha (TNF-α), which are central mediators of the hyperinflammatory response observed in severe COVID-19 (50). Moreover, phagocytosis of SARS-CoV-2-infected apoptotic cells by macrophages has been shown to activate plasmacytoid dendritic cells (pDCs), leading to enhanced secretion of type I interferons and TNF, further amplifying the immune response (51).

In summary, the functional convergence of “Endocytosis” (KEGG:04144), “Fc gamma receptor (FcγR)-mediated phagocytosis” (KEGG:04666), and “Phagosome maturation” (KEGG:04145) constitutes a coherent mechanistic axis exploited by SARS-CoV-2 to mediate cellular entry, circumvent innate immune defenses, and potentiate dysregulated inflammatory responses. These pathways are intricately interconnected, with key molecular effectors, such as CD32 (encoded by FCGR2A), serving overlapping roles across multiple nodes within this triad and within the broader COVID-19-associated interactome. The integrity and regulation of each pathway are critical determinants of viral pathogenesis, influencing processes ranging from endocytic uptake and intracellular trafficking to antigen degradation and cytokine production. Consequently, targeted modulation of endosomal dynamics, FcγR signaling, or phagosomal processing offers a rational therapeutic approach to attenuate viral replication, enhance immunological clearance, and mitigate host tissue injury driven by hyperinflammatory states. This integrated framework highlights the importance of host-pathogen interface pathways as both facilitators of infection and potential targets for host-directed therapies in COVID-19.

### Conclusions

In conclusion, our comprehensive analysis of RNA modifications during SARS-CoV-2 infection reveals a multifaceted role for epitranscriptomic regulation in both viral pathogenesis and the host’s response.

Although the present study is primarily descriptive, the identified RNA modification patterns suggest several mechanistic implications for host–virus interactions. Altered m6A deposition may regulate the stability and translation efficiency of both host and viral transcripts, influencing viral replication and innate immune signaling. Similarly, m5C modifications mediated by NSUN family methyltransferases are known to modulate RNA export, translation, and immune recognition, thereby shaping antiviral responses. The increased pseudouridylation observed in infected samples may enhance RNA structural stability and translational capacity while reducing activation of pattern-recognition receptors, representing a potential viral evasion strategy. Collectively, these findings indicate that SARS-CoV-2 infection induces a coordinated remodeling of the host epitranscriptome that could influence viral persistence and immune activation, warranting further functional studies to validate these predicted molecular effects.

The observed positional and sequence-specific distribution of these modifications suggests a tightly controlled epitranscriptomic response to infection. Specifically, m5C modification, primarily mediated by NSUN2, functions as a host restriction factor. It destabilizes viral RNAs and modulates key immune-related genes, thereby influencing both viral replication and immune activation. Similarly, dynamic changes in m6A methylation impact critical signaling pathways and immune functions, underscoring the complexity of RNA modification networks during infection. Furthermore, alterations in pseudouridylation contribute to translational control and immune modulation, with significant implications for viral evasion and the efficacy of mRNA vaccines. Moreover, the study revealed significant enrichment in KEGG pathways—“Endocytosis” (KEGG:04144), “FcγR-mediated phagocytosis” (KEGG:04666), and “Phagosome” (KEGG:04145)—provide a systems-level perspective on the molecular interplay underlying SARS-CoV-2 pathogenesis. Their coordinated activation and dysregulation highlight critical intervention points for therapeutic strategies aimed at disrupting viral entry, modulating immune responses, and limiting COVID-19-associated immunopathology. Collectively, these findings highlight the critical importance of RNA modifications as potential targets for therapeutic intervention. Further mechanistic studies are warranted to fully elucidate their precise roles in SARS-CoV-2 biology.

### Study limitations

It should be noted that the identification of RNA modification sites in this study is based on computational prediction using CHEUI and NanoSPA. While both approaches have been benchmarked against experimentally validated data sets and exhibit high predictive accuracy, the results presented here remain inferential. Consequently, the reported lists of modified genes and loci should be interpreted as putative high-confidence candidates requiring further biochemical validation. Future experimental approaches such as m6A- or m5C-specific immunoprecipitation, bisulfite conversion, or pseudouridine profiling will be essential to confirm these predictions and elucidate their functional relevance in SARS-CoV-2 infection.

This study was performed on human clinical material, which limits the feasibility of direct experimental validation such as gene silencing or mutagenesis. Nevertheless, by combining computational detection tools with biological pathway analysis, we provide a high-confidence atlas of RNA modifications associated with SARS-CoV-2 infection. These findings serve as a starting point for future mechanistic work in model systems to assess the functional significance of the identified epitranscriptomic changes.

## Data Availability

The authors confirm that all data generated and analyzed during this study are either included in this published article or available from the corresponding authors upon reasonable request. The data underlying this article are available in the European Nucleotide Archive repository, under accession numbers PRJEB84380 and PRJEB74103.

## SUPPLEMENTAL MATERIAL

Supplemental tables spectrum.02561-25-s0001.xlsx Tables S1 to S11.

ASM does not own the copyrights to Supplemental Material that may be linked to, or accessed through, an article. The authors have granted ASM a non-exclusive, world-wide license to publish the Supplemental Material files. Please contact the corresponding author directly for reuse.
